# Ultrasound features of medullary thyroid carcinoma correlate with cancer aggressiveness: a retrospective multicenter study

**DOI:** 10.1186/s13046-014-0087-4

**Published:** 2014-10-25

**Authors:** Pierpaolo Trimboli, Luca Giovanella, Stefano Valabrega, Massimiliano Andrioli, Roberto Baldelli, Nadia Cremonini, Fabio Rossi, Leo Guidobaldi, Agnese Barnabei, Francesca Rota, Antonella Paoloni, Laura Rizza, Giorgio Fattorini, Maurizio Latini, Claudio Ventura, Paolo Falasca, Fabio Orlandi, Anna Crescenzi, Ferdinando D’Ambrosio, Vito Cantisani, Francesco Romanelli, Roberto Negro, Enrico Saggiorato, Marialuisa Appetecchia

**Affiliations:** Section of Endocrinology and Diabetology, Ospedale Israelitico, Rome, Italy; Department of Nuclear Medicine and Thyroid Centre, Oncology Institute of Southern Switzerland, Bellinzona, Switzerland; Department of Medical and Surgical Sciences, Ospedale S. Andrea, Sapienza University, Rome, Italy; EndocrinologiaOggi, Rome, Italy; Endocrinology Unit, Regina Elena National Cancer Institute, Rome, Italy; U.O.C. Endocrinology, Ospedale Maggiore, Azienda USL, Bologna, Italy; Section of Pathology, Ospedale Israelitico, Rome, Italy; Department of Experimental Medicine, Sapienza University, Rome, Italy; Internal Medicine, Ospedale S. Sebastiano Martire of Frascati, Rome, Italy; Section of Endocrinology, Division of Internal Medicine, Department of Clinical and Biological Sciences, University of Turin, Torino, Italy; Pathology Unit, Campus Bio-Medico University Hospital, Rome, Italy; Department of Radiology, Oncology and Anatomo Pathology, Sapienza University, Rome, Italy; Division of Endocrinology, Ospedale V. Fazzi, Lecce, Italy; Service d’Endocrinologie, Pôle de Médecine, Centre Hospitalier des Escartons, Briançon, France; Ospedale Israelitico di Roma, Via Fulda, 14, 00148 Rome, Italy

**Keywords:** Medullary thyroid cancer, Ultrasonography, Histology, Thyroid nodule

## Abstract

**Background:**

Poor prognosis of medullary thyroid cancer (MTC) with suspicious ultrasound (US) features has been reported. The aim of the study was to investigate the association between preoperative US presentation and aggressiveness features of MTC. Also, US features of MTC were compared with those previously reported.

**Methods:**

Study group comprised 134 MTC from nine different centers. Based on US presentation the nodules were stratified in “at risk for malignancy” (m-MTC) or “probably benign” (b-MTC) lesions.

**Results:**

Eighty nine (66.4%) m-MTC and 45 (33.6%) b-MTC were found. Metastatic lymph nodes (p = 0.0001) and extrathyroid invasiveness (p < 0.0001) were more frequent in m-MTC. There was statistically significant correlation (p = 0.0002) between advanced TNM stage and m-MTC with an Odds Ratio 5.5 (95% CI 2.1–14.4). Mean postsurgical calcitonin values were 224 ± 64 pg/ml in m-MTC and 51 ± 21 in b-MTC (p = 0.003).

**Conclusions:**

This study showed that sonographically suspicious MTC is frequently associated with features of aggressiveness, suggesting that careful preoperative US of MTC patients may better plan their surgical approach.

## Introduction

Medullary thyroid cancer (MTC) originates from thyroid C cells and accounts for about 5% of thyroid malignancy [1]. MTC may occur as sporadic tumor (about 80% of cases) or be part of a familial disorder [1]. The diagnosis of MTC represents a diagnostic challenge in clinical practice. Fine needle aspiration (FNA) of thyroid nodules has several pitfalls for this histologic type. The cytologic examination can diagnose MTC with classical presentation, and the detection rate was reported of 56% in a recent meta-analysis [2]. The routine measurement of serum calcitonin is still a matter of debate and ultrasonography (US) does not achieve high reliability rates [3]. Due to these limitations, many MTC are still incidentally discovered after thyroid excision, leading to the risk of an incomplete therapeutic approach and thus of a poorer prognosis [4]. To diagnose MTC prior to surgery is of high importance. This allows to examine other disorders potentially associated with hereditary forms of MTC and increases the possibility to achieve a complete surgical cure. Therefore, a carefully planned initial surgical treatment of patients with the preoperative diagnosis of MTC is strongly required [1].

Ultrasound examination is the pivotal imaging tool in the risk stratification of thyroid nodules. It allows the identification of non palpable nodules and the assessment of their characteristics. Several papers have reported the presence of specific US features as highly suggestive of malignancy [1,5]. Moreover, preoperative neck US evaluation is the gold standard in the surgical planning of patients undergoing thyroidectomy [5]. However, most studies have focused on the US features of differentiated papillary thyroid carcinoma (PTC), and only limited data are available regarding the US criteria for possibly malignant MTCs, and the possible association between US features of MTCs and cancer aggressiveness [6–12].

The aim of this study was investigate the aggressiveness features of MTC in association with their preoperative US presentation. The US features of MTC in comparison to those reported in the literature are also described.

## Materials and methods

This multicenter retrospective study included patients who had been diagnosed and operated for MTC over the period from March 2007 to March 2013 at nine different centers. The preoperative diagnosis had been based on high serum calcitonin levels with a suggestive cytology and/or detection of calcitonin in fine needle aspiration washout [13–15]. All patients had undergone total thyroidectomy with central nodal neck dissection in all cases. Patients with suspicious neck lymph nodes on preoperative imaging had undergone lateral neck dissection. In all patients the diagnosis of MTC was confirmed by histology according to the WHO classification criteria [16]. Tumour staging was based on the TNM classification [17]. Postoperative parameters that could be associated with aggressiveness including the presence of lymph node involvement (pN1), extrathyroid tumor extension, RET mutation, multifocality of lesions, concomitant C cell hyperplasia and MEN 2 were recorded by reviewing the patients files.

The US appearance of the lesions was assessed by retrieving and reviewing the preoperative thyroid and neck US images in the institution PACS systems. In order to assess the risk of malignancy by US, all nodules were assessed by four reviewers with more than ten years experience in thyroid US (PT, ES, VC, LuGi) according to a previously described validated classification system. This classification system stratifies nodules in classes 1 to 5 with intermediate steps of 0.5 for classes 2 to 5 and nodules with category 3.5 or greater are regarded as probably malignant with a positive predictive value of 97% [18,19]. Briefly, class 1 includes round or oval anechoic lesion, in class 2 there are regular-shaped nodules with cystic change, class 3 contains solid and regular-shaped nodule, class 4 comprises solid and regular-shaped nodule, while solid and irregular-shaped nodules with extrathyroid extension are in class 5. Based on this system nodules with class ≥3.5 were categorized as “malignant” (m-MTC) and nodules with class <3.5 as “indeterminate or benign” (b-MTC). Discordant cases of the present study were categorized by the examiners in consensus. In case of multifocal MTC, only the most prominent focus was analyzed in the study.

Statistical analysis was performed using standard statistical software using Graph Pad Prism (Graph Pad Software Inc, La Jolla, CA, USA). Differences in frequencies were analyzed by chi-square test or Fisher exact test and differences in mean values were evaluated using t-test. Statistical significance was set at p < 0.05. Means and standard errors were compared using Mann-Whitney test. The association of suspicious US features of MTC with TNM stages was analyzed using Odds Ratio (OR).

## Results

The study group consisted of 134 patients (85 females, 49 males, mean age 56.7 ± 1.2 years) with histologically proved MTC. In 10.4% of cases there was a familial MTC. Tumors were unifocal in 122/134 (91%) patients and multifocal in 12/134 [9]. Concomitant C cell hyperplasia was found in 6/134 (4.5%) cases and MEN 2 in 2/134 (1.5%) patients. The clinical, biochemical and histologic characteristics of the study group are displayed in Table 1.

**Table 1:** **Clinical characteristics of the study group**

Age of patients (years)*	56.7 ± 1.2
Preoperative calcitonin (pg/ml)*	558 ± 62
Postoperative calcitonin (pg/ml)*	167 ± 44
Nodule’s size (mm)*	19.8 ± 1.0
C cell hyperplasia *(number of patients/total)*	6/134
MEN 2 *(number of patients/total)*	2/134
Hereditary cancer *(number of patients/total)*	14/134
Single nodule *(number of patients/total)*	72/134

Footnote: *Mean values ± Standard Deviation.

Based on the US criteria for risk evaluation, 89/134 (66.4%) were classified as m-MTC and 45/134 (33.6%) as b-MTC. There was no statistically significant difference between the two groups regarding the lesion size (m-MTC, 19.7 ± 1.3 mm; b-MTC, 20.1 ± 1.7, t-test p = 0.13) and patients’ age (m-MTC, 56.9 ± 1.5 years; b-MTC, 56.6 ± 2.4 years, t-test p = 0.37).

Involvement of neck lymph nodes (p = 0.0001) and extrathyroid tumor extension (p < 0.0001) were significantly more frequent in m-MTC than in b-MTC (Table 2). Concomitant C cell hyperplasia and MEN 2 were recorded only in m-MTC. More than one preoperative parameter of tumor aggressiveness was significantly more present m-MTCs than b-MTC (p < 0.0001) (Table 2).

**Table 2:** **Comparison of predictors of tumor aggressiveness**

**Parameter**	**m-MTC (n = 89)**	**b-MTC (n = 45)**	***p*** **value**
pN1 status	43 (48.3%)	5 (11.1%)	0.0001
Extrathyroid tumor extension	27 (30.3%)	1 (2.2%)	<0.0001
RET mutation	12 (13.4%)	2 (4.4%)	0.13
C cell hyperplasia	6 (6.7%)	0	0.09
Multifocal lesions	11 (12.3%)	1 (2.2%)	0.06
Associated MEN-2	2 (2.2%)	0	0.55
Presence of >1 of the above parameters	61 (68.5%)	8 (17.8%)	<0.0001
Detectable postoperative serum CT	42.2%	20%	0.04

*Abbreviations*: pN1 status: neck lymph node involvement, MEN: multiple endocrine neoplasia, m-MTC: medullary thyroid cancer with “malignant” US appearance; b-MTC: medullary thyroid cancer with “benign or indeterminate” US appearance.

**Figure 1:**
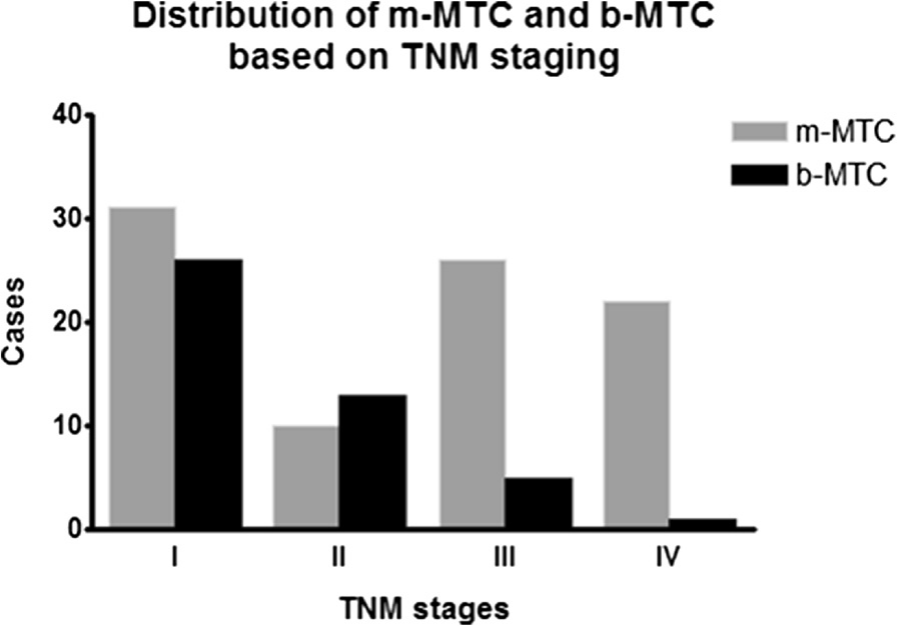
**TNM 2010 staging (17) of 134 histologically proved medullary thyroid cancers with “malignant” (m-MTC) or “benign or indeterminate” (b-MTC) ultrasound presentation.**

Advanced TNM stage (III-IV) was significantly (p < 0.0001) more frequent in m-MTC (48 cases, 53.9%) than in b-MTC (6 cases, 13.3%). Suspicious US appearance (m-MTC) was significantly (p = 0.0002) associated with TNM stages III-IV with an OR of 5.5 (95% CI 2.1–14.4) (Figure 1).

Preoperative serum calcitonin levels were available in 117 cases. Significant difference in mean preoperative calcitonin levels was found (595 ± 76 pg/ml in m-MTC, 484 ± 106 pg/ml in b-MTC p = 0.0001). Mean postoperative calcitonin value was available in 106 patients, 30/71 (42.2%) m-MTC and 7/35 (20%) b-MTC cases had detectable calcitonin. Mean postoperative calcitonin value was significantly (p = 0.003) higher in the m-MTC group (224 ± 64 pg/ml) compared to b-MTC group (51 ± 21 pg/ml).

## Discussion

In the last decade, high resolution US has been widely available and this contributed to an increase in the diagnosis of thyroid malignancy [20,21]. The presence of specific US features has been closely associated with higher risk for malignancy [22]. Most of the US data in the literature concern the papillary thyroid cancers, because of its higher frequency among thyroid malignancies (about 80%) [20,21], whereas limited and discordant data are available about the US characteristics of medullary carcinomas [6–11].

**Figure 2:**
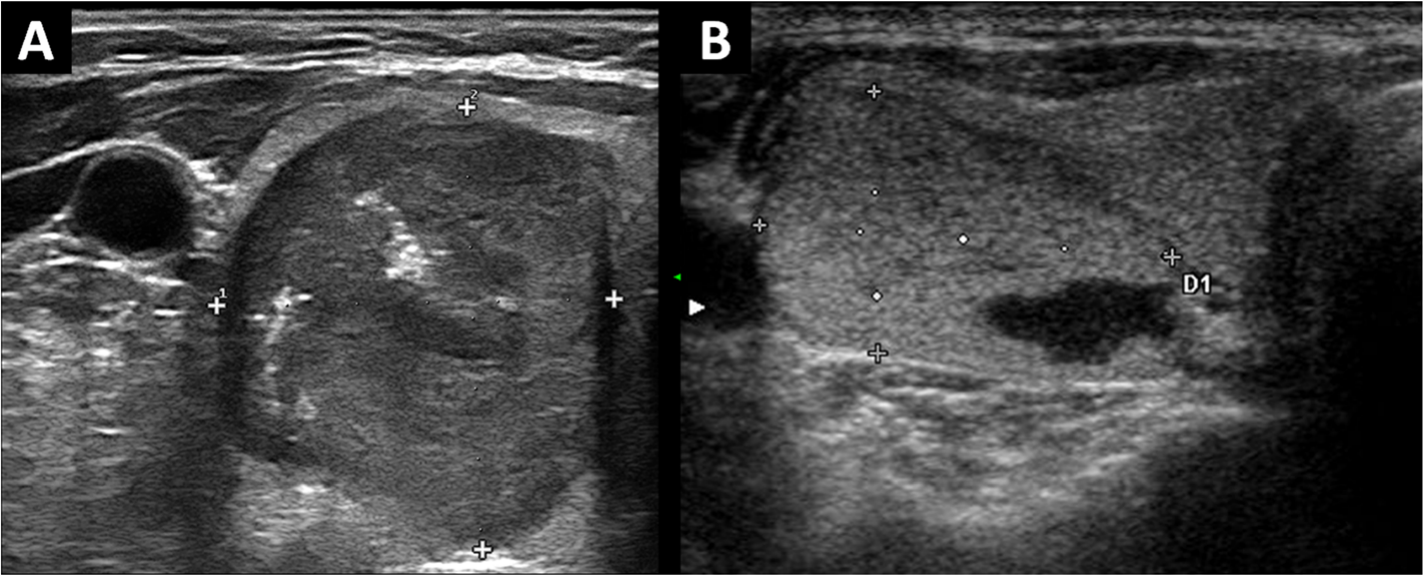
**Different ultrasound presentations of MTC. (A)** hypoechoic nodule with calcifications, classified at ultrasonography as suspicious. **(B)** mixed-spongiform nodule with hypoechoic halo, non-suspicious at ultrasonography.

The aim of this study was to investigate the association between the features suggesting MTC aggressiveness and its preoperative US features by reviewing the MTC cases diagnosed in nine different centers over the last six years. An amount of 66% of MTC could be classified as “at-risk” by ultrasound examination, while a not negligible part (34%) was not ultrasonographically suspicious (Figure 2). As the main finding, a suspicious US presentation of MTC conferred a 450% increase of risk of advanced TNM stages. Furthermore, the presence of characteristics of higher tumor aggressiveness was significantly more frequent in the group with a “malignant” ultrasonographic presentation. In addition, the value of serum calcitonin and before and after surgery was significantly higher in m-MTC group. Present data suggest that m-MTC behave as a more aggressive tumor. As the first one, Fukushima et al [12] studied this topic analyzing the prognosis of medullary thyroid cancer in correlation with “benign” or “malignant” US presentation. That interesting series was collected over nineteen years (1988–2007) and included only nonhereditary cancers. Of the 77 cases, 70% were malignant at US (“M-type”) and 30% benign (“B-type”). The “B-type” MTC were highly indolent tumors and had excellent prognosis with significant postsurgical drop of both calcitonin; on the contrary, the “M-type” MTC were associated with neck lymph nodes involvement, extrathyroid invasion and biochemical persistence of disease. Here we used the same ultrasound risk stratification adopted by Fukushima and colleagues [12], and our results perfectly agree with that data. Furthermore, present study explains the results reported by Fukushima et al [12]. Based on both series, a preoperative ultrasound examination should be performed in all MTC patients to better tailor the surgical approach. In clinical practice these data achieve high importance. In fact, MTC prognosis depends on extent of disease at diagnosis, presence of regional lymph node metastases, completeness of the surgical resection and undetectable postsurgical calcitonin [1,23].

As a second objective, here we analyzed the US reliability in diagnosing MTC lesions and our data might be discussed in comparison to the previous reports [6–10]. In all, these papers reported that a not negligible percentage of MTC may be found with no ultrasound risk features. In particular, characteristics of benignancy, such as round shape, cystic changes, homogeneous echostructure, and circumscribed margins, were often recorded in MTC. More recently, these data confirm the previous experience of one center of those participant to the present multicenter study; there, MTC and PTC were compared with a large benign control group, and MTC showed poor discrepancy with respect to the controls [11]. Also, a heterogeneous elastographic presentation was recently described [24]. Here we recorded that about one in three MTC nodules manifests at ultrasonography as benign, being this finding quite similar to that reported by Fukushima et al [12]. This finding extends the conclusions of all the above studies [6–12] and indirectly prompts to use calcitonin measurement in the initial evaluation of thyroid nodules [2,4]. In general, the possibility to submit to FNA all nodules with a significant size (i.e. 1 cm) should be taken into account, even if they have a “benign” US presentation.

The strength of this paper is the large sample size of MTC and the multicenter design. Also, it has to be underlined that the series was collected in a recent period using high resolution ultrasound systems which improved both diagnosis and follow-up of several conditions [22,24–26]. To date, the knowledge on US presentation of MTC is poor, and the present data strongly improve the literature on this matter.

In conclusion, this study showed that MTC with preoperative “at-risk” US presentation is more frequently associated with features of tumor aggressiveness than those with no suspicious ultrasonography. In clinical practice, these data suggest a preoperative thyroid ultrasound assessment of MTC patients to better plan their surgical approach. Further prospective studies on this topic are necessary.
